# Prevalence and prognosis of non-specific chest pain among patients hospitalized for suspected acute coronary syndrome - a systematic literature search

**DOI:** 10.1186/1741-7015-10-58

**Published:** 2012-06-12

**Authors:** Vidar Ruddox, Mariann Mathisen, Jan Erik Otterstad

**Affiliations:** 1 Department of Cardiology, Vestfold Hospital Trust, Tønsberg, Norway; 2 Medical Libraries, Vestfold Hospital Trust, Tønsberg, Norway

**Keywords:** Hospitalizations, non-specific chest pain, non-cardiac chest pain, atypical chest pain, chest pain not yet diagnosed, acute coronary syndrome, prognosis, readmissions, mortality

## Abstract

**Background:**

The term non-specific chest pain (NSCP) is applied to hospitalized patients in order to designate that they neither have an acute coronary syndrome (ACS) nor display evidence of a coronary ischemia. The number of NSCP patients is increasing and comprehensive guidelines specifying their optimal management have not yet been introduced. The objective of this review was to explore the prevalence and prognosis of NSCP versus ACS among patients recruited in consecutive series hospitalized for chest pain suspected to be ACS.

**Methods:**

This is a systematic literature search where three databases were searched from 1990 to 14 November 2011. In addition, one database was searched for Epub ahead of print per 24 March 2012. Three inclusion criteria were applied: 1. documentation of an unselected consecutive series of patients admitted for chest pain, where this review is based upon two groups of patients defined as follows: a) 'ACS/high-risk' and b) NSCP; 2. at least 100 cases with NSCP; and 3. follow-up of hospital readmissions and mortality for at least six months.

**Results:**

A total of 2,204 citations were screened after removal of duplicates. Out of 80 full text articles assessed for eligibility 12 studies were included, comprising 24,829 patients (inter-study range 250 to 13,762), with 11,008 (44%) categorized as NSCP and 13,821 (56%) as 'ACS/high-risk'. The mean one-year total mortality rate among patients with NSCP in nine studies was 3.2% (inter-study range 1.4% to 8.1%), with the highest mortality among patients with pre-existing coronary heart disease (CHD). The mean one-year mortality rate among 'ACS/high-risk' patients was 18.0% (inter-study range 14.0% to 19.9%) in four studies with available data. In six studies the mean one-year readmission rate for patients with NSCP was 17.5% (inter-study range 2.5% to 40%).

**Conclusions:**

Patients with NSCP represent a large, heterogeneous and important group. Due to co-existing CHD in nearly 40% of these patients, their prognosis is not necessarily benign. Although their average one-year mortality rate was almost six times lower than those with 'ACS/high-risk', the subset with concomitant CHD had a relatively poor prognosis when compared with NSCP patients without evidence of CHD.

## Introduction

At present, early invasive management and optimal medical treatment of patients with acute coronary syndrome (ACS) is well established. Many patients admitted to hospital with chest pain suggestive of ACS, however, do not fulfil criteria for such management due to normal cardiac markers and/or no objective evidence of ischemia. The term 'non-specific chest pain' (NSCP), among many others (for example, non-cardiac chest pain, atypical chest pain, chest pain not yet diagnosed), has been introduced in order to describe the subset of patients without coronary ischemic etiology of their chest pain [1-4]. At present, there are no comprehensive guidelines for optimal management of NSCP. A substantial number of these patients may have pre-existing coronary heart disease (CHD) and many tend to be readmitted for similar symptoms [5]. Accordingly, NSCP patients account for a significant amount of hospital resources. The European Society of Cardiology has recently stated that a large number of patients classified as low risk for an ACS (where patient history, physical examination, electrocardiogram (ECG) and cardiac biomarkers are not diagnostic) represent the most prevalent group of patients admitted to hospital with chest pain, and, thus, are the most challenging of chest pain patients [6].

The objective of this review is to obtain information regarding the prevalence and prognosis of NSCP in comparison with patients with ACS. Such information might be of importance in the optimal management of patients with NSCP since there are no guidelines for this important and large group. In order to minimize selection bias, we searched only for studies of consecutive, unselected patients admitted to hospital with chest pain suspected to be ACS.

## Methods

The review protocol is presented in Appendix A [see Additional file 1]. We searched the following three electronic databases: EMBASE, MEDLINE and PsycINFO from the year 1990 onward to 14 November 2011. In addition we included a PubMed search for Epub articles ahead of print as per 24 March 2012. The search strategies combined text words and subject headings identifying reports relating to ACS, non-specific chest pain (NSCP, that is, chest pain not associated with current criteria for ACS or obvious non-coronary reasons). All citations were checked for duplications both electronically (Reference Manager) and manually. We also performed highly specific searches for the incidence rate and prognosis of NSCP, and to this end we continuously scanned reference lists of included studies for additional citations. Citations from reference lists considered of adequate quality were included as 'additional citations identified through other sources'.

Eligible studies needed to state that consecutive patients were admitted with chest pain in order to rule out ACS. Only English language original articles were accepted for inclusion. Reviews, meta-analyses, population-based studies, abstract-only, case reports and letters to the editor were not considered eligible.

Two investigators (VR and JEO) independently reviewed all citations in order to identify potentially relevant articles and resolved any discrepancies by reaching a consensus. If two or more studies presented the same data from a single patient population then only one was included.

Due to expected differences in the definition of ACS, we introduced the term 'ACS/high-risk' for patients assumed to have ischemic chest pain. All other patients were defined as NSCP provided no severe non-coronary etiology was identified such as pulmonary disorders (pneumonia, pleuritis, embolism, and pneumothorax), myo-pericarditis, aortic dissection and obvious, severe gastrointestinal disorders.

Whenever feasible, an attempt was made to subdivide the NSCP group into those with established coronary heart disease (history of ACS, percutaneous coronary intervention (PCI), coronary artery bypass graft (CABG) and angina, preferably with a positive exercise test and/or positive findings on coronary angiography) and patients without evidence of CHD, termed CHD +/- respectively.

The following inclusion criteria were applied:

1. Documentation of an unselected consecutive series of patients admitted to hospital with chest pain suspected to be ACS. This implies that studies requiring informed consent for follow-up data were not included.

2. An arbitrarily chosen figure of at least 100 patients with NSCP was required to avoid small studies and any subsequent type 2 statistical errors.

3. Long-term follow-up on readmissions and/or mortality had to be at least six months after the index hospitalization in both subgroups.

4. Although desirable, studies without follow-up data on 'ACS/high risk' patients could be included provided the other inclusion criteria were met.

## Results

### Study selection

**Figure 1:**
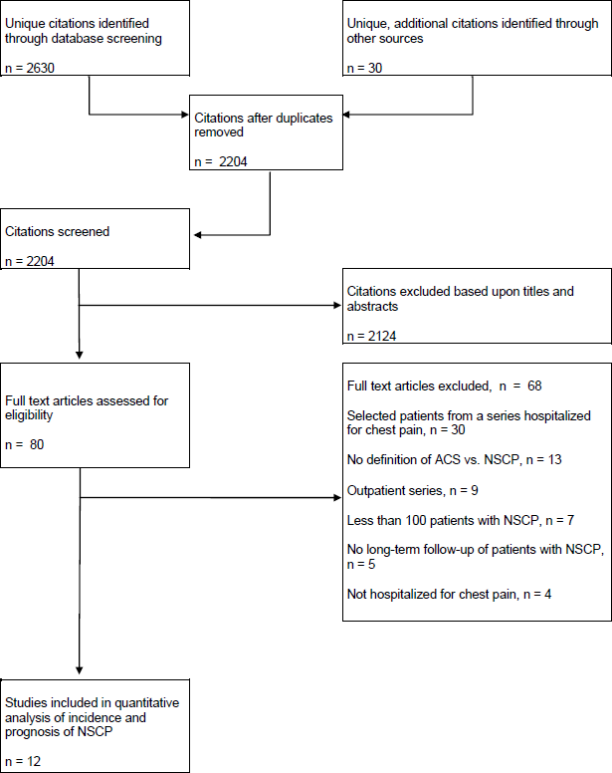
**Selection of studies Flow of information obtained from a systematic search of four databases (PUBMED, MEDLINE, EMBASE and PsycINFO) with addition of citations identified through other sources, and presentation of full text articles that have been evaluated and excluded for predefined reasons**. ACS, acute coronary syndrome; NSCP, non-specific chest pain.

A flow chart of the different phases in the study selection is presented in Figure 1. After adding additional citations and eliminating duplicates, 2,204 citations were screened and 80 full articles were selected for assessment. The reasons for excluding 68 of these are also listed in Figure 1. References are grouped according to the reasons for exclusion given in Appendix B [see Additional file 2]. Among the 12 accepted articles (Table 1), three were additional citations identified through other sources and one was found among ahead of print citations.

**Table 1:** Incidence and prognosis of nonspecific chest pain among 24,829 patients admitted to hospital to rule out acute coronary syndrome.

				NSCP			Follow-up of NSCP patients			
**Study**	**Year**	**n**	**ACS/high risk** **n (%)**	**CHD+**	**CHD-**	**Total** **n (%)**	**n**	**Time**. **month**	**Mortality** **n (%)**	**Readmissions** **n (%)**

Panju (7)	1996	1,235	1,077 (87)	12	146	158 (13)	100	36	3 (3)	19 (19)
Bholashing (8)^a^	2001	2,271	791 (35)	n.a.	n.a.	1,480 (65)	653	24	16 (2.5)^b^	n.a.
Cassin (9)	2002	570	224 (39)	n.a.	n.a.	346 (61)	266	12	4 (1.5)	n.a.
Edmon (10)	2002	423	269 (64)	59	95	154 (36)	152	12	5 (3.3)	33 (22)
Conti (11)	2002	13,762	9,335 (68)	n.a.	n.a.	4,427 (32)	870	6	0	n.a.
Menown (12)	2003	391	196 (50)	158	37	195 (50)	195	12	8 (4)	46 (18)
Spalding (13)	2003	250	142 (57)	24	84	108 (43)	103	12	3 (2.9)	14 (14)
Shaver (14)	2004	999	307 (31)	n.a.	n.a.	692 (61)	692	12	n.a.	277 (40)
Aune (15)^a^	2006	755	366 (48)	164	225	389 (52)	389	12	22 (5.7)	n.a.
Aune (16,17)^a^	2010	934	363 (39)	203	368	571 (61)	571	12	18 (3.2)	n.a.
Leise (18)	2010	1,973	1,608 (82)	n.a.	n.a.	356 (18)	320	240	90 (28)	157 (49)
Ravn-Fischer (20)	2011	1,266	234 (18)	n.a.	n.a.	1,032 (82)	234	12	84 (8.1)	n.a.

Mortality and readmission percentages have been converted to annual rates. ^a^Citations were not within the systematic search; ^b^cardiac, not total, death rate. ACS, acute coronary syndrome; CHD, coronary heart disease; n, number; NSCP, Non-specific chest pain

The included studies were published between 1996 and 2012 and together include 24,829 patients. Information on previous CHD among patients with NSCP is provided in six studies, long-term mortality for patients with NSCP in ten and for patients with 'ACS/high risk' in four studies. Data on readmissions was available for NCSP in six studies. Only one study reported readmission rates for 'ACS/high risk'. These data are presented in Table 1.

### Background information on the studies presented in table 1

In Panju *et al.*'s study [7] patients were admitted to the Coronary Care Unit (CCU). Those with either acute myocardial infarction (AMI) or unstable angina pectoris (UAP) were categorized as 'ACS/high-risk', with the remaining patients categorized as NSCP in this review. Due to co-morbidity and/or unwillingness only 100 NSCP patients were followed up for three years in terms of mortality and readmission. Their one-year mortality and readmission rates were 3% and 19%, respectively.

Bholasingh *et al. *[8] included 2,271 patients from two cohorts presenting with chest pain to the cardiac emergency room in 1994 and 1996. The 'ACS/high-risk group' comprised of patients admitted to the CCU, and the rest, categorized as NSCP, were discharged after normal CK-MB measurements. In the two cohorts combined 653/1,480 NSCP patients who remained under observation in the cardiac emergency room were followed up in terms of cardiac mortality for two years. The authors observed that in all 16 patients who experienced a fatal cardiac event during follow-up CHD was either documented before the index hospitalization or was established as the index hospitalization discharge diagnosis. Annual cardiac mortality rate was 1.25%.

Cassin *et al. *[9] studied patients admitted to the Emergency Department (ED) for acute chest pain, with patients categorized to 'ACS/high-risk' according to well defined criteria for high-risk. The one-year mortality rate among the 346 patients classified as NSCP was 1.5%.

In Edmon *et al.*'s [10] study of the efficacy and safety of a chest pain pathway patients not deemed suitable for the pathway were admitted directly to the CCU and categorized as 'ACS/high-risk'. The remaining patients were designated to the pathway and represented the NSCP group. The one-year mortality rate among these 154 patients was 3.3% and the readmission rate 22%.

In Conti *et al.*'s study [11] consecutive patients with chest pain admitted to the ED were screened. Based upon a validated chest pain score patients with intermediate and high-risk were categorized as 'ACS/high-risk'. Among the remaining patients forming the NSCP group, 1,755 with a chest pain score ≥4 were submitted to a short-stay program in the CCU for further evaluation. CHD was documented in 885 of these cases. The six-month mortality among the remaining 870 patients showing no evidence of CHD was 0%. For comparison, the six-month mortality rates among 2,420 patients with AMI and 3,764 with UAP were 10.6% and 1.1%, respectively.

Menown *et al. *[12] studied consecutive patients with ischemic-type chest pain admitted to hospital in order to follow the prognostic impact of various markers assessed in two separate blood samples. The 'ACS/high-risk' group comprised of patients with elevated levels of CK-MB or troponins. Among the remaining patients categorized as NSCP, 81% had suspected/known CHD. Their one-year mortality rate was 4% as opposed to 14% in the 'ACS/high risk' group selected on the basis of cardiac marker elevation. The one-year readmission rate of NSCP patients was 18%.

Spalding *et al. *[13] included patients admitted either to the CCU or medical assessment unit with chest pain suspected to be of cardiac origin. The one-year mortality rate among patients with NSCP (termed atypical chest pain) was 2.7%, compared with 18.3% among 'ACS/high risk' patients (termed acute ischemic event). One-year readmission rate of patients with NSCP was 14%.

Shaver *et al. *[14] studied patients admitted to hospital in order to evaluate potential ACS. The 'ACS/high-risk' category consisted of patients who either had a positive test for underlying CHD, had a final diagnosis of UAP or AMI, or who died in hospital. Long-term follow-up data comprised a high readmission rate (40%) for the 692 patients categorized as NSCP. Mortality data is not provided for any of the two groups of patients.

Two cohorts studied by Aune *et al. *comprised otherwise unselected patients admitted to hospital for chest pain in 2003 [15] and 2006 [16,17]. The diagnostic criteria for ACS were identical for both cohorts. Patients with overt non-coronary co-morbidities explaining their chest pain were excluded. The percentage of NSCP increased from 52% in 2003 to 61% in 2006 [17]. The respective percentages of pre-existing CHD among patients with NSCP were 41% in 2003 and 35% in 2006. In the two cohorts combined the one-year mortality rate among the 960 patients with NSCP was 4.2%, with 8.4% in 367 patients with pre-existing CHD versus 1.5% in 593 patients without CHD. The one-year mortality rate in 729 patients in the 'ACS/high risk' group was 19.9%.

In Leise *et al.*'s study [18] patients were admitted to the ED with chest pain, of whom 95% were admitted to hospital. Patients categorized as 'ACS/high-risk' were sent home from the hospital with a diagnosis of chest pain related to CHD or specific cardiac disease, the remaining were categorized as having NSCP. The NSCP 20-year mortality rate was 28%, indicating an average of 1.4% fatalities per year. This mortality rate was not significantly different from an expected survival curve created for the Minnesota white population in the period 1950-2000. In an adjusted analysis for other covariates, only age, Charlsons Co-morbidity Index (severity-weighted index of co-morbid conditions) [19], previous CABG and previous valvular disease were significant predictors for all-cause mortality among the patients with NSCP. The readmission rate of patients with NSCP was 49% over 20 years, indicating, on average, 2.5% per year.

The study of Ravn-Fiscer *et al. *[20] comprised all patients hospitalized with acute chest pain in three hospitals within the Sahlgrenska University of Gothenburg. The 'ACS/high risk' group comprised patients with ST-segment elevation myocardial infarction (STEMI; n = 79), and UAP/non-ST-segment myocardial infarction (NSTEMI); n = 155). The remaining patients were categorized as NSCP (termed non-ACS group). The one-year mortality rate in the former group was 19.7%, as opposed to 8.1% in the latter. No information on readmissions is provided for any subgroup.

### Summary of results

In total, 11,008 (44%) patients were categorized as having NSCP according to the criteria applied in the present review. The prevalence of NSCP ranged between 18% and 82% in the studies selected. In six studies with available information on whether pre-existing CHD was present or not, 620/1,575 (39%) were CHD+ as opposed to 955/1,575 (61%) CHD-. In six studies the mean one-year readmission rate for patients with NSCP was 17.5% (inter-study range 2.5% to 40%).

In the NSCP group, the mean one-year mortality rate in nine studies was 3.2% (range 1.4 to 8.1%), excluding the study of Conti *et al. *[11] which had a six month cardiac mortality rate of 0% and the study of Bholasingh *et al. *[8] in which cardiac, not total, mortality was reported. Only one study reported the mortality rate for NSCP patients both with and without concomitant CHD, this being 8.4% and 1.5%, respectively [17].

For patients in the 'ACS/high-risk' groups the mean one-year mortality rate was 18.0% (inter-study range 14% to 19.9%) in the four studies where this was reported. One study reported a six month mortally rate of 10.6% for patients with AMI and 1.1% with UAP [11]. None of the selected studies provided readmission rates for the 'ACS/high risk' group.

## Discussion

### Summary of evidence

Patients with NSCP represent a large and heterogeneous group of patients hospitalized for chest pain. Compared with 'ACS/high risk' patients they have a good long-term prognosis. A substantial number of these patients, however, have previously known CHD, and according to the findings of Aune *et al *[15-17] such patients have a one-year mortality within the range of both those who have been treated with primary PCI for STEMI and those patients with UAP. The rates of readmissions were high, reflecting the significant burden of health costs represented by these patients.

The introduction of more sensitive assays for troponins combined with new sensitive markers, such as Copeptin [21] and high sensitive Troponin T [22], will probably convert a number of non-ACS patients into patients with NSTEMI. Accordingly, some patients with NSCP may have potential benefit from early invasive management and intense medical treatment in cases where there are positive markers. On the other hand, the relatively poor specificity may give rise to the overtreatment of patients with non-thrombotic causes where the rise of cardiac markers can be seen.

In the two cohort studies of Aune *et al.*, NSCP patients without CHD admitted to the ED increased from 30% of all patients hospitalized for chest pain in 2003 to 39% in 2006 [15-17]. With the same cut-off levels for Troponin T, the proportion of NSCP with CHD remained unchanged (22% in both cohorts). Although NSCP patients without CHD have low one-year mortality, quality of life may be influenced by their symptoms. There is at present no good evidence by which to characterize and quantify those who end up as 'frequent flyers' (FF).

The subset of NSCP patients with co-existing CHD may be more concerned about pain that is of non-cardiac origin, which may not worry others without a history of CHD. Nevertheless, both groups need a careful evaluation of other possible etiologies for their non-ischemic chest pain. Furthermore, as Zaman *et al. *[23] point out, there are differences in the way chest pain is reported both among the sexes and different ethnic groups. Atypical chest pain was, according to their material, reported by more women than men and by more South Asian people than white people. They found that typical symptoms of stable angina were associated with coronary outcomes in all patients and that prognostically important angina did not present atypically in women and South Asians. This implies that white men, but indeed all patient categories, presenting with chest pain in general should be considered both for careful cardiac and non-cardiac assessments. Based upon the academic literature, many patients with NSCP may potentially profit from management of psychiatric, gastrointestinal or musculoskeletal disorders [7,11,13,18,24-27]. Although not apparent from studies exploring the etiology of NSCP, it seems reasonable that several patients may represent combinations of these disorders, with need for various interventions. As recently reported by White *et al. *[27], psychiatric etiologies for NSCP may include both alexithymia ('no words for feelings') and increased anxiety sensitivity, each requiring two quite different psychological interventions. The importance of an association between psychiatric disorders and NSCP has recently been verified in a population-based cohort study form Scotland [28]. Individuals with a hospital discharge diagnosis of NSCP who have a previous psychiatric hospitalization have a greater risk of death, all-cause, and cardiovascular-specific, at one year, than those without. The authors suggest that a NSCP hospitalization is an opportunity to engage and, where appropriate, intervene to modify cardiovascular risk in this difficult-to-reach and high-risk patient group.

At present there is no consensus on how to manage NSCP patients. The European Society of Cardiology (ESC) has recently advocated the widespread use of Chest Pain Units (CPU) to which patients are admitted following a rapid triage in the ED [6]. The CPU enables proper monitoring and further continuous evaluation of chest pain patients, including repeat ECG tracings, cardiac biomarker blood drawings and cardiac imaging. Such patients should have their evaluation completed within 24 hours in order that they be discharged safely or relocated to the cardiology department for medical therapy and/or coronary angiography. Considering a number of the possible etiologies of NSCP, a CPU might also include a standardized investigational program with 'fast tracks' to non-cardiac assessments of unexplained patient chest pain. Potential gastrointestinal, psychiatric and musculoskeletal disorders may be managed and hopefully be of value to the patients' quality of life. Such management may be of value, since these patients with NSCP might have high morbidity (for example, recurrent admissions) that might be improved by focusing away from the heart and more on other causes of their pain. If patients are simply reassured and discharged with a negative troponin test, they will, given a lack of explanation, simply come back again the next time they experience pain in order to have a troponin done. So far, such an additional approach to systematically evaluate possible non-cardiac causes of chest pain not been incorporated into the ESC statement.

### Risk of bias in the review

The diagnostic criteria for ACS and 'high-risk' patients have an influence upon the risk level among the remaining patients with NSCP. These diagnostic criteria have been changed and refined throughout the time period this search covers. The prevalence of established CHD and co-morbidity in NSCP patients is important for their prognosis in the respective studies.

The selection criteria for patients recruited from consecutive series of patients hospitalized for chest pain should minimize, but not eliminate, selection bias. In the USA the majority of patients are admitted to the ED or CPU, whereas in Europe patients are admitted 'to hospital', CCUs or ICUs. In the 12 studies listed, patients were admitted to 'the hospital' in five, to 'the ED' in three, to 'the CCU in one, to the 'CCU or medical assessment unit' in one, to the 'cardiac emergency room in one and to a 'CPU' in one. This bias reflects in the fact that seven studies were from Europe, three from the USA, one from Canada and one from New Zealand. The risk level in these studies is inevitably associated with the hospital scenario on admission. Patients selected for admission to a CCU will have a higher risk than the broad spectrum of patients admitted to an ED. Furthermore, the criteria applied for the 'ACS/high-risk' group are quite heterogeneous across studies. During the time period covered in this review the diagnostic criteria for AMI and ACS has changed considerably. It is probable that a significant number of patients with a diagnosis of NSCP in the 1990s would have had an AMI diagnosis in more recent years. Both bias in scenario and criteria for the high-risk group can explain the wide ranges observed for the incidence and prognosis of NSCP. The importance of sub-dividing NSCP patients into CHD+ and CHD- is especially important according to the studies of Aune *et al. *[15-17], which show a nearly six-fold increase of one-year mortality in the former group.

### Limitations

Apart from the obvious selection bias both within and across studies, a systematic search is not without problems. Important studies with more extensive and uniform results may well have fallen outside the search criteria. Indeed, three of the 12 studies included in this review were not found within the systematic search made. In order to discover the studies by Aune *et al. *[15-17] the search would have to be expanded to include more than 100,000 citations. Several excellent studies have not been included in the review for predefined reasons, the most frequent being more or less selected study populations (Appendix B).

No attempts were made to explore studies of the causes of NSCP or the characteristics of so-called FF patients. Present information on the causes of NSCP and the proper characteristics of FFs is fairly incomplete. Future studies should, therefore, aim towards a careful characterization of all NSCP, including a standardized diagnostic approach. Studies with a prospective definition of an FF with follow-up of readmissions could be helpful in identifying those prone to ending up as a FF versus those who do not.

## Conclusions

Patients with NSCP represent a large, heterogeneous and important group. Due to co-existing CHD in nearly 40% of these patients, their prognosis is not necessarily benign, but still far better than those with ACS. The introduction of new markers and sensitive essays for cardiac necrosis may reduce the problematic subset of non-ACS patients with CHD and instead convert them to a diagnosis of ACS with optimal management. The remaining patients may be offered a comprehensive non-cardiac evaluation of their symptoms in order to explore possible causes of their pain.

This review has highlighted the general shortcoming of NSCP studies; a considerable selection bias within and across studies. Guidelines for the management of patients with NSCP are necessary.

## Abbreviations

ACS: acute coronary syndrome; AMI: acute myocardial infarction; CABG: coronary artery bypass graft; CCU: coronary care unit; CHD: coronary heart disease; CPU: chest pain unit; ECG: electrocardiogram; ED: Emergency Department; ESC: European Society of Cardiology; FF: frequent flyer; NSCP: non-specific chest pain; NSTEMI: non-ST-segment myocardial infarction; PCI: percutaneous coronary intervention; STEMI: ST-segment elevation myocardial infarction; UAP: unstable angina pectoris.

## Competing interests

The authors declare that they have no competing interests.

## Authors' contributions

This study was conceived and designed by VR, MM and JEO. The literature search was performed by MM. VR and JEO independently reviewed all titles and abstracts to identify potentially relevant articles. VR and JEO analyzed and interpreted the data. VR and JEO drafted the original version of the manuscript and revised it for critically important intellectual content. All authors have read and approved the final manuscript.

## Pre-publication history

The pre-publication history for this paper can be accessed here:

http://www.biomedcentral.com/1741-7015/10/58/prepub

## Supplementary Material

Additional file 1 **Appendix A - The search strategy**. Appendix A presents the full search strategy of the following three electronic databases: EMBASE, MEDLINE and PsycINFO from the year 1990 onward to 14 November 2011. In addition a PubMed search for Epub articles ahead of print as per 24 March 2012 is presented. The search strategies combine text words and subject headings identifying reports relating to ACS, non-specific chest pain. Click here for file

Additional file 2 **Appendix B - Rejected Citations**. Full citations evaluated and rejected according to the prospectively defined inclusion criteria. References are grouped according to the reasons for exclusion. Click here for file
