# Supplementary material for: Prevalence and prognosis of non-specific chest pain among patients hospitalized for suspected acute coronary syndrome - a systematic literature search
Source: BMC Med. 2012 Jun 12;10:58. doi: 10.1186/1741-7015-10-58 (PMC3391179; doi:10.1186/1741-7015-10-58)
Supplement: Additional file 1 — Appendix A - The search strategy. Appendix A presents the full search strategy of the following three electronic databases: EMBASE, MEDLINE and PsycINFO from the year 1990 onward to 14 November 2011. In addition a PubMed search for Epub articles ahead of print as per 24 March 2012 is presented. The search strategies combine text words and subject headings identifying reports relating to ACS, non-specific chest pain. [file 1741-7015-10-58-S1.DOC]

**Appendix A - Search Strategy**

Database(s): **Embase** 1988 to 2011 Week 45**, Ovid MEDLINE(R) In-Process & Other Non-Indexed Citations and Ovid MEDLINE(R)** 1948 to Present**, PsycINFO** 1987 to November Week 2 2011
Search Strategy:

| **#** | **Searches** | **Results** |
| --- | --- | --- |
| 1 | thorax pain/ | 32117 |
| 2 | noncardiac chest pain/ | 286 |
| 3 | syndrome X/ | 1506 |
| 4 | (chest burn or ((cardiac or chest or heart or thorax or thoracic) adj2 pain) or ((cardiac or angina) adj2 syndrome x) or (microvascular adj2 angina) or ((suspected or potential or subsequent* or recurrent or repeat* or previous*) adj2 (acute coronary syndrome* or acs or acute myocardial infarct* or ami or stroke*))).tw. | 60664 |
| 5 | or/1-4 | 79234 |
| 6 | hospitalization/ | 202412 |
| 7 | statistics/ | 182340 |
| 8 | 6 and 7 | 6571 |
| 9 | *hospital utilization/ | 351 |
| 10 | "length of stay"/ | 104432 |
| 11 | *hospital admission/ | 8858 |
| 12 | hospital discharge/ | 46501 |
| 13 | hospital readmission/ | 14061 |
| 14 | "hospital subdivisions and components"/ | 5618 |
| 15 | 8 and 14 | 35 |
| 16 | *health care utilization/ | 14863 |
| 17 | (ecu visit* or re-admi* or readmi* or re-hospital* or rehospital* or previous* hospitali?* or (frequent* adj1 (use* or attend* or fl?er*)) or (health adj3 (care or service*) adj3 (consum* or seeking behavio?r* or use* or utili?ation*)) or ((number or rate*) adj3 (admission* or visit*)) or (repeat* adj2 visit*) or ((subsequent* or recurrent or repeat* or previous*) adj2 hospital adj2 (admission* or encounter* or visit*)) or (patient* adj2 discharge*)).tw. | 204214 |
| 18 | or/8-13,15-17 | 353006 |
| 19 | 5 and 18 | 4301 |
| 20 | limit 19 to "causation-etiology (maximizes sensitivity)" [Limit not valid in PsycINFO; records were retained] | 2621 |
| 21 | et.fs. | 3525638 |
| 22 | 19 and 21 | 716 |
| 23 | 20 or 22 | 2933 |
| 24 | incidence/ or prevalence/ | 704769 |
| 25 | cross-sectional study/ | 192393 |
| 26 | (incidence* or prevalence* or epidemiol*).tw. | 1881312 |
| 27 | ep.fs. | 1717731 |
| 28 | or/24-27 | 3120653 |
| 29 | 19 and 28 | 979 |
| 30 | outcome assessment/ | 142611 |
| 31 | fatality/ | 44131 |
| 32 | mortality/ | 378222 |
| 33 | survival rate/ | 215704 |
| 34 | treatment outcome/ | 1061509 |
| 35 | (cardiac adj2 outcome*).tw. | 5249 |
| 36 | or/30-35 | 1702119 |
| 37 | 19 and 36 | 1192 |
| 38 | limit 19 to "prognosis (best balance of sensitivity and specificity)" [Limit not valid in PsycINFO; records were retained] | 1765 |
| 39 | 37 or 38 | 2371 |
| 40 | 23 or 29 or 39 | 3501 |
| 41 | cohort analysis/ | 235185 |
| 42 | follow up/ | 513363 |
| 43 | longitudinal study/ or prospective study/ or retrospective study/ | 1189303 |
| 44 | ((prospective or retrospective) adj3 stud*).tw. | 609662 |
| 45 | (consecutiv* adj5 patient*).tw. | 275631 |
| 46 | or/41-45 | 2134718 |
| 47 | 40 and 46 | 1728 |
| 48 | limit 47 to (danish or english or german or norwegian or swedish) | 1619 |
| 49 | limit 48 to yr="1990 -Current" | 1585 |
| 50 | 49 use emed | 1085 |
| 51 | Chest Pain/ | 40050 |
| 52 | Microvascular Angina/ | 2335 |
| 53 | (chest burn or ((cardiac or chest or heart or thorax or thoracic) adj2 pain) or ((cardiac or angina) adj2 syndrome x) or (microvascular adj2 angina) or ((suspected or potential or subsequent* or recurrent or repeat* or previous*) adj2 (acute coronary syndrome* or acs or acute myocardial infarct* or ami or stroke*))).tw. | 60664 |
| 54 | or/51-53 | 82127 |
| 55 | Hospitalization/sn, ut [Statistics & Numerical Data, Utilization] | 13597 |
| 56 | "length of stay"/ or patient admission/ or patient discharge/ or patient readmission/ or patient transfer/ | 260692 |
| 57 | Health Services/ut [Utilization] | 5503 |
| 58 | Recurrence/ | 244288 |
| 59 | Hospital Units/sn, ut [Statistics & Numerical Data, Utilization] | 737 |
| 60 | (ecu visit* or re-admi* or readmi* or re-hospital* or rehospital* or previous* hospitali?* or (frequent* adj1 (use* or attend* or fl?er*)) or (health adj3 (care or service*) adj3 (consum* or seeking behavio?r* or use* or utili?ation*)) or ((number or rate*) adj3 (admission* or visit*)) or (repeat* adj2 visit*) or ((subsequent* or recurrent or repeat* or previous*) adj2 hospital adj2 (admission* or encounter* or visit*)) or (patient* adj2 discharge*)).tw. | 204214 |
| 61 | or/55-60 | 679034 |
| 62 | 54 and 61 | 9891 |
| 63 | limit 62 to "causation-etiology (maximizes sensitivity)" [Limit not valid in PsycINFO; records were retained] | 5943 |
| 64 | etiology.fs. | 1886143 |
| 65 | 62 and 64 | 1246 |
| 66 | 63 or 65 | 6447 |
| 67 | incidence/ or prevalence/ | 704769 |
| 68 | Cross-Sectional Studies/ | 192393 |
| 69 | (incidence* or prevalence* or epidemiol*).tw. | 1881312 |
| 70 | epidemiology.fs. | 1025376 |
| 71 | or/67-70 | 2767509 |
| 72 | 62 and 71 | 2065 |
| 73 | "Outcome Assessment (Health Care)"/ | 183577 |
| 74 | fatal outcome/ or hospital mortality/ or survival rate/ | 613696 |
| 75 | treatment outcome/ | 1061509 |
| 76 | (cardiac adj2 outcome*).tw. | 5249 |
| 77 | mortality.fs. | 361199 |
| 78 | or/73-77 | 1984493 |
| 79 | 62 and 78 | 3179 |
| 80 | limit 62 to "prognosis (maximizes sensitivity)" [Limit not valid in PsycINFO; records were retained] | 7072 |
| 81 | 79 or 80 | 7661 |
| 82 | 66 or 72 or 81 | 8891 |
| 83 | cohort studies/ or longitudinal studies/ or follow-up studies/ or prospective studies/ or retrospective studies/ | 2082864 |
| 84 | ((prospective or retrospective) adj3 stud*).tw. | 609662 |
| 85 | (consecutiv* adj5 patient*).tw. | 275631 |
| 86 | or/83-85 | 2444199 |
| 87 | 82 and 86 | 3907 |
| 88 | limit 87 to (danish or english or german or norwegian or swedish) | 3648 |
| 89 | limit 88 to yr="1990 -Current" | 3510 |
| 90 | 89 use prmz | 1374 |
| 91 | angina pectoris/ | 59797 |
| 92 | chest pain.id. | 258 |
| 93 | (chest burn or ((cardiac or chest or heart or thorax or thoracic) adj2 pain) or ((cardiac or angina) adj2 syndrome x) or (microvascular adj2 angina) or ((suspected or potential or subsequent* or recurrent or repeat* or previous*) adj2 (acute coronary syndrome* or acs or acute myocardial infarct* or ami or stroke*))).tw. | 60664 |
| 94 | or/91-93 | 114995 |
| 95 | hospitalization/ or hospital admission/ or hospital discharge/ | 306369 |
| 96 | treatment duration/ | 66275 |
| 97 | client transfer/ | 129 |
| 98 | (Readmission or hospital admission or (Health adj (care or service) adj utilization)).id. | 2907 |
| 99 | (ecu visit* or re-admi* or readmi* or re-hospital* or rehospital* or previous* hospitali?* or (frequent* adj1 (use* or attend* or fl?er*)) or (health adj3 (care or service*) adj3 (consum* or seeking behavio?r* or use* or utili?ation*)) or ((number or rate*) adj3 (admission* or visit*)) or (repeat* adj2 visit*) or ((subsequent* or recurrent or repeat* or previous*) adj2 hospital adj2 (admission* or encounter* or visit*)) or (patient* adj2 discharge*)).tw. | 204214 |
| 100 | or/95-99 | 542810 |
| 101 | 94 and 100 | 7845 |
| 102 | limit 101 to (danish or english or german or norwegian or swedish) | 6992 |
| 103 | limit 102 to yr="1990 -Current" | 6725 |
| 104 | 103 use psyf | 71 |
| 105 | 50 or 90 or 104 | 2530 |
| 106 | remove duplicates from 105 | 2074 |

**Frequent flyers – PubMed – Epub ahead-of-print – 2012-03-24**

History

| **Search** | **Query** | **Items found** |
| --- | --- | --- |
| [#5](http://www.ncbi.nlm.nih.gov.proxy.helsebiblioteket.no/pubmed/advanced) | Select 100 document(s) | [100](http://www.ncbi.nlm.nih.gov.proxy.helsebiblioteket.no/pubmed/?cmd=HistorySearch&querykey=5) |
| [#4](http://www.ncbi.nlm.nih.gov.proxy.helsebiblioteket.no/pubmed/advanced) | Search (#1) AND #2 Limits: published in the last 2 years | [1063](http://www.ncbi.nlm.nih.gov.proxy.helsebiblioteket.no/pubmed/?cmd=HistorySearch&querykey=4) |
| [#3](http://www.ncbi.nlm.nih.gov.proxy.helsebiblioteket.no/pubmed/advanced) | Search (#1) AND #2 | [5756](http://www.ncbi.nlm.nih.gov.proxy.helsebiblioteket.no/pubmed/?cmd=HistorySearch&querykey=3) |
| [#2](http://www.ncbi.nlm.nih.gov.proxy.helsebiblioteket.no/pubmed/advanced) | Search (“ecu visit*” OR re-admi* OR readmi* OR “re-hospital*” OR rehospital* OR “previous* hospitaliz*” OR “previous* hospitalis*” OR “frequent* use*” OR “frequent* attend*” OR “frequent flier*” OR “frequent flyer*” OR ((“health care service*”) AND (consum* OR “seeking behavior*” OR “seeking behaviour*” OR use* OR utilization* OR utilisation*)) OR ((number OR rate*) AND (admission* OR visit*)) OR (repeat* AND visit*) OR ((subsequent* OR recurrent OR repeat* OR previous*) AND hospital AND (admission* OR encounter* OR visit*)) OR “patient discharge*” OR “patients discharge*”) | [130845](http://www.ncbi.nlm.nih.gov.proxy.helsebiblioteket.no/pubmed/?cmd=HistorySearch&querykey=2) |
| [#1](http://www.ncbi.nlm.nih.gov.proxy.helsebiblioteket.no/pubmed/advanced) | Search (“chest burn” OR “cardiac pain” OR “chest pain” OR “heart pain” OR “thorax pain” OR “thoracic pain” OR “cardiac syndrome x” OR “angina syndrome x” OR “microvascular angina” OR ((suspected OR potential OR subsequent* OR recurrent OR repeat* OR previous*) AND (“acute coronary syndrome*” OR acs OR “acute myocardial infarct*” OR ami OR stroke*))) | [111424](http://www.ncbi.nlm.nih.gov.proxy.helsebiblioteket.no/pubmed/?cmd=HistorySearch&querykey=1) |
| [#0](http://www.ncbi.nlm.nih.gov.proxy.helsebiblioteket.no/pubmed/advanced) | pubmed clipboard | [100](http://www.ncbi.nlm.nih.gov.proxy.helsebiblioteket.no/pubmed/clipboard) |
