# Supplementary material for: Prevalence and prognosis of non-specific chest pain among patients hospitalized for suspected acute coronary syndrome - a systematic literature search
Source: BMC Med. 2012 Jun 12;10:58. doi: 10.1186/1741-7015-10-58 (PMC3391179; doi:10.1186/1741-7015-10-58)
Supplement: Additional file 2 — Appendix B - Rejected Citations. Full citations evaluated and rejected according to the prospectively defined inclusion criteria. References are grouped according to the reasons for exclusion. [file 1741-7015-10-58-S2.DOC]

**Appendix B – Rejected Citations**

**Full citations evaluated and rejected (citations in the search)**

**Selected patients from a series of patients hospitalized for chest pain, n = 30**

- Viswanthan K, Kilcullen N, Morrel C, Thislehtwaite SJ, Sivananthan Mu, Hassan TB, Barth JH. Heart-type fatty acid-binding protein predicts long-term mortality and reinfarction in consecutive patients who are troponin-negative. J Am College Cardiol 2010 55: 2590-98.*
- Dagnone E, Collier C, Pickett W, Ali N, Miller M, Tod D, Morton R. Chest pain with nondiagnostic electrocardiogram in the emergency department: a randomized controlled trial of two marker regimens. Canad Med Assoc J 2000; 162: 1561-66.
- Wyrick JJ, Kalvaitis S, McConnel J, Rinkevich D, Kaul S, Wei K. Cost efficiency of myocardial contrast echocardiography in patients presenting to the emergency department with chest pain of suspected cardiac origin and a non-diagnostic electrocardiogram. Am J Cardiol 2008; 102: 649-52.
- Bholasingh R, Cornel JH, Kamp O, van Straalen JP, Sanders GT, Dijkmsman L, Tijssen JGP, de Winter RJ. The prognostic value of markers of inflammation in patients with troponin T-negative chest pain before discharge from the emergency department. Am J Med 2003; 115: 521-28.*
- Farkouh ME, Smars PA, Reeder GS, Zinsmeier AR, Evans RW, Meloy TD, Kopecky SL, Allen M, Allison TG, Gibbons RJ, Gabriel SE. A clinical trial of a chest-pain observation unit for patients with unstable angina. New Engl J Med 1998; 339: 1882-88.
- Mayou RA, Thompson DR. Treatment needs of patients admitted for acute chest pain. J Psychosomatic Res 2002; 53: 1177-83.*
- Mayou RA, Gill D, Thompson DR, Day A, Hicks N, Volmink J, Neil A. Depression and anxiety as predictors of outcome after myocardial infarction. Psychosomatic Med 2000; 62: 212-19.*
- Sanchis J, Bodì V, Nùnes J, Bosch X, Loma-Osorio P, Mainar L, Santas E, Milfania G, Robles R, Llàcer À. Linitations of clinical history for evaluation of patients with acute chest pain, non-diagnostic electrocardiogram, and normal troponin. Am J Cardiol 2008; 10: 613-17.
- Madsen T, Mallin M, Bledsoe J, Bossart P, Davis V, Gee C, Barton E. Utility of the emergency department observation unit in ensuring stress testing in low-risk chest pain patients. Crit Pathways in Cardiol 2009; 8: 122-24.*
- Grace SL, Abbey SE, Irvine J, Shnek ZM, Stewart DE. Prospective examination of anxiety persistence and its relationship to cardiac symptoms and recurrent events. Psychoter Psychosom 2004; 73: 344-62.*
- Beigel R, Oieru D, Goitein O, Chouraqui P, Feinberg MS, Brosh S Asher Em, Konen E, Shamiss A, Eldar M, Hod H, Or J, Matetzky S, Fast track evaluation of patients with acute chest pain: experience in a large-scale chest pain unit in Israel. IMAJ 2010; 12: 329-33.*
- Nerenberg RH,Shofer FS, Robey JL, Brown AM, Hollander JE. Impact of a negative prior stress test on emergency physician disposition in ED patients with chest pain syndromes.. Am J Emerg Med 2007; 25: 30-44.*
- Frasure-Smith N, Lespèrance F, Gravel G, Marsson A, Juneau M, Bourassa MG. Long-term survival differences among low-anxious, high-anxious and repressive copers enrolled in the Montreal Heart Attack Readjustment Trial. Psychosomatic med 2002; 64: 571-79.
- Madsen T, Mallin M, Bledsoe J, Bossart P, Davis V, Gee C, Barton E. Utility of the emergency department observation unit in ensuring stress testing in low-risk chest pain patients. Crit Pathways in Cardiol 2009; 8: 122-24.
- Sekhri N, Feder C, Junghans C, hemingway H, Timmis AD. How effective are rapid access chest pain clinica? Prognnosis of incident angina and non-cardiac chest pain in 8762 consecutive patients. Heart 2007; 93: 458-463.
- Fagring AJ, Lappas G, Kjellgren KI, Welin C, Manhem K, Rosengren A. Twenty-year trends in incidence and 1-year mortality in Swedish patients hospitalized with non-AMI chest pain. Data from 1987-2006 from the Swedissh hospital and death registries. Heart 2010; 96: 1043-49.
- Sicari R, Rigo F, Cortigani L, Gherardi S, Galderisi M, Picano E. Additive prognostic value of coronary flow reserve in patients with chest pain syndrome and normal or near-normal coronary arteries. Am J Cardiol 2009; 103: 626-631.
- Jaffery Z, Nowak R, Khoury N, Tokarski G, Lanfear D, Jacobsen G, McCord J. Myoglobin and troponin I elevation predict 5-year mortality in patients with undifferentiated chest pain in the mergency department. Am Heart J 2008; 156: 939-45.
- Gorelik O, Almoznino-Sarafian, Yarovoy, I, Alon I, Shteinshnaider M, Charchashvily, Modai D, Cohen N: Patient-related variables predicting acute coronary syndrome following admission for chest pain of possible coronary origin. Coronary art dis 2008; 17:15-21*
- Herlitz J, Karlson BW, Sjölin M. Re-admissions among patints with acute chest pain who were discharged from the meregncy department. Eur J Emerg Med 1996; 3: 31-3
- Fruegaard P, Launbjerg B, Hesse B, Jørgensen F, Petri A, Eiken P, Aggestrup P, Elsborg L, Mellemgaard K. The diagnoses of patienys admitted with chest pain but without myocardial infarction. Eur Heart J 1996; 17: 1028-1034. (not in search)
- Karlsson BW, Sjöland H, Währborg P, Lindquist J, Herlitz J. Patients discharged from emergency care after acute myocardial infarction was rudeld out: early follow-up in relation to gender. Eur J Emerg Med 1997; 4: 72-80.
- Coley KC, Saul MI, seybert AL. Economic burden of not recognizing panic disorder in the emergency department. J Emerg Med 2009; 36: 3-7.
- Sanchez M, Lôpez B, Bragulat E, Gòmez-Angelats E, Jimenez S, Ortega M, Coll-Vincent B, Miro O. Predictors and outcomes of frequent chest pain unit users. Am J Emerg Med 2009; 27: 660-67.
- Eslick GD, Talley NJ. Natural history and predictors of outcome for non-cardiac chest pain: a prospective 4-year cohort study. Neurogastroenterol Motil 2008; 20: 989-997
- Nabi F, Chang SM, Xu Jiaqiong, Gigliotti E, Mahmarian JJ. Assessingrisk in acute chest pain: The value of stress myocardial perfusion imaging in patients admitted through the mergency department. J Nucl Cardiol 2012; 19: 233-43.
- Conti A, Poggioni C, Viviani G, Luzzi, M, Vicidomini S, Zanobetti M, Innicenti F, Pini R, Padeeletti L, Gensini GF. Short- and long-term cardiac events in patientswith chest pain with or without known existing coronary disease presenting normal electrocardiogram. Am J Emerg Med 2012 Mar 16 (Epup ahead of print)
- Scheuermeyer FX, Innes G, Grafstein E, kiss M, Boyhcuk B, Yu E, Kalla D, Christianson J. Safety and efficacy of a chest pain diagnostic algorithm with selective outpatient stress testing for emergency department patients with potential ischemic chest pain. Ann Emerg Med 2012; 59: 256-64.
- Singer AJ, Domingo A, Thode HC, Daubert M, Vainrib AF, Ferraro S, Minton A, Poon A, Henry M. Utilization of coronary computed tomography angiography for exclusion of coronary artery disease in ED patients woth low- to intermediate risk chest pain: a 1 year experience. Am J Emerg Med 2012 Mar 16 (Epub ahead of prtint).
- McKinley S, Fien M, Meischke H, AbiRuz Mohammad E, Lennie TA, Moser DK. Complicatiobs after acute coronary syndrome are reduced by perceived control of cardiac illness. J advanced nursing 2011 Jan 11 (Epub ahead of print)

**No definition of ACS vs. NSCP, n = 13**

- Madsen T, Bossart P, Bledsoe J, Bernhisel K, Cheng M, Mataoa T, Barlett J, McKellar A, Rivas W, Quick N. Patients with coronary disase fail observation status at higher rates than patients without coronary disease. Am J Emerg Med 2010; 28: 19-22.*
- Stenerstrand U, Wijkman M, Fredrikson M, Nyström FH, Association between admission supine systolic blood pressure and 1-year mortality in patients admitted to the intensive care unit for acute chest pain. JAMA 2010; 303: 1167-72.*
- Martinez-Sellès, Bueno H, Sacristàn A, Estèvez A, Ortiz J, Gallego L, Fernàndez-Avilès F. Chest pain in the emergency department: Incidence, clinical characteristics, and risk stratification. Rev Esp Cardiol 2008; 61: 953-59.*
- Hollander JE, Robey JL, Chase MR, Brown AM, Zogby KE, Schofer FS. Relationship between a clear-cut alternative diagnosis and 30-day outcome in emergency department patients with chest pain. Acad Emerg Med 2007; 14: 210-15.*
- Brennan M-L, Penn MS, Van Lente FV, Nambi V, Shisehbor MH, Aviles RJ et al. Prognostic value of myeloperixidase in patients with chest pain. New Engl J Med 2003; 349: 1595-604.
- Solinas L, Raucci R, Terrazino S,Moscariello F, Pertoldi F, Vajto S, Badano L. Prevalence, clinical characteristics, resource utilization and outcome of patients with acute chest pain in the emergency department. A multicenter, prospective, observational study in North-Eastern Italy. Ital Heart J 2003; 4: 318-24.*
- Ng SM, Krishnawaswamy, Morissey R, Clapton P, Fitzegerald R, Maisel AS. Ninety-minute accelerated critical pathway for chest pain evaluation. A, J Cardiol 2001; 88: 611-17.*
- Christenson J, Innes G, McKnight D, Boychuk B, Grafstein E, Thompson CR, Rosenberg F, Anis AH, Gin K, Tilley J, Wong H, Singer J. Safety and efficiency of emergency department assessment of chest discomfort. CMAJ 2004; 170: 1803-07.*
- Herlitz J, Karlson BW, Lindquist J, Sjölin M. Important factors for the 10-year mortality rate in patients with acute chest pain or other symptoms consistent with acute myocardial infarction with particular emphasis on the influence of age. Am Hear J 2001; 42: 624-32.
- Ohlsson-Önerud Å, Svensson L, Szecsödy P, S¨derberg Anne Charlotte, Nordlander R. Bröstsm¨rtenhet – bra alternativ vid låg risk för kardial genes. Läkartidn 2002; 99: 4848-53.
- Wallman R, Llorca J, Gòmez-Acebo I, Ortega Castellanos O, Roldan Rojo F, Dierssen-Sotos. Prediction of 30-day cardiac-related-emergency-readmissions using simple administrative hospital data. Int J Cardiol 2011 Jul 18 (Epub ahread of print).
- Dang Thang N, Karlson BW, Bergman B, Santos M, Karlsson T, Bengtson Ann, Johanson P, Rawshani A, Herlitz J. Patients admitted to hospital with chest pain – changes in a 20-year perspective. Int J Cardiol 2011 Nov 7 (Epub ahead of print).
- Birkham RH, Wen T, Datilo PA, Briggs WM, Parekh A, Arkun A, Byrd B, Gaeta TJ. Improving patients flow in acute coronary syndromes in the face og hospital crowding. J emerg med 2011 =ct 19 (Epub ahead of print).

**Outpatient series, n = 9**

- Dammen T, Arnesen H, Ekeberg Ø, Friis S. Psychological factors, pain attribution and medical morbidity in chest-pain patients with and without coronary artery disease. Gen Hosp Psychiatry 2004; 26: 463-69.
- Spinhoven P, Van der Does AJW, Van Dijk E, Van Rood YR. Heart focused anxiety as a mediating variable in the treatment of noncardiac chest pain by cognitive-behavioral therapy and paroxetine. J Psychosomat Res 2010; 69: 227-33.
- Dammen T, Bringager CB, Arnesen H, Ekeberg Ø, Friis S. A 1-year follow-up study of chest pain patients with and without panic disorder. Gen Hosp Psychiatry 2006; 28: 516-24.*
- White KS, Raffa SD, Jakle KR, Stoddard JA, Barlow DH, Brown TA, Covino NA. Morbidity of *DSM-IV* Axis I disorders in patients with noncardiac chest pain: psychiatric morbidity linked with increased pain and health care utilization. J Consult and Clin Psychology 2008; 76: 422-30.*
- Budzynski J. Exertional esophageal pH-metry and manometry in recurrent chest pain. World J Gastroenterol 2010; 14: 4305-12.
- Ågård A, Bentley L, Herlitz J. Experience and concerns among patients being treated for atypical chest pain. Eur J Int Med 2003; 16: 339-44.
- Chen Y-H, Chen S-F, Lin H-C, Lee H-C. Healthcare utilization patters before and after contact with psychiatrist care for panic disorder. J Affective Disorders 2009; 119: 173-76.
- Bringager VB, Gauer K, Arnesen H, Friis S, Dammen T. Nonfearful disorder in chest-pain patients: status after nine-year follow-up. Psychosomatics 2008; 49: 426-37.*
- Mayou R, Bryant B, Forfar C, Clark D. Non-cardiac chest pain and benign palpitations in the cardiac clinic. Br Heart J 1994; 72: 548-53.*

**Less than 100 patients with NSCP, n = 7**

- Tew R, Guthrie EA, Creed FH, Cotter L, Kisely S, Tomenson B. A long-term follow-up study of patents with ischaemic heart disease versus patients with nonspecific chest pain. J Psychosomatic Res 1995; 39: 977-85.
- Garcia-Campayo J, Rosei F, Serrano P, Santed MA, Andrès E, Roca M, Serrano-Bianco, Latre ML. Different psychological profiles in non-cardiac chest pain and coronary artery disease: a controlled study. Rev Esp Cardiol 2010; 63: 357-61.
- Kisely SR, Russel EM, Creed FH. Psychological aspects of recurrent chest pain. J Royal Soc Med 1993; 86: 516-18.*
- Soares-Filho GLF, Freire RC, Biancha K, Pacheco T, Volschan A, Valenca A, Nardi AE. Use of the hospital anxiety and depression scale (HADS) in a cardiac emergency room – chest pain unit. Clinics 2009; 64: 209-14.
- Eifert GH, Hodson SE, Tracey DR, Seville JL, Gunawardene K. Heart-focused anxiety, illness beliefs, and behavioral impairment: comparing healthy heart-anxious patients with cardiac and surgical inpatients. J Behavioral Med 1996; 19: 385-98.
- Smith K, Ross D, Connoly E. Investigating 6-month health outcomes of patients with angina discharged from a chest pain service. Eur J Cardiovac Nursing 2002; 1: 253-64.*
- Metcalfe MJ, Rawles JM, Schirreffs C, Jennings K. Six year follow up of a consecutive series of patients presenting to the coronary care unit with acute chest pain: prognostic importance of the electrocardiogram. Br Heart J 1990; 267-72.

**No long-term follow-up of patients with NSCP, n = 5**

- Udvarhely S, Goldman L, Komaroff L, Lee T. determinats of resource utilization for patients admitted for evaluation of acute chest pain. J Gen Intern Med 1992; 7: 1 – 10.*
- Durand E, Delos A, Chaib A, Lepillier A, Beretti S, Collin M, et al. Performance assessment of a chest pain unit: Preliminary 2-year experience in the European Georges Pompidou Hospital. Arch Cardiovasc Dis 2009; 102: 801-9.*
- Abdul-Mohsen MF, Al-Quorain, Al-Hamdan AA, Husain A, Qutur; Lapido GOA. Clinical profile of patients admitted to the coronary care unit with possible myocardial infarction without diagnostic ECG and/or enzyme changes. East African Med J. 1993; 70: 777-81.*
- Pope JH, Aufderheide TP, Ruthazer R, Woolard RH, Feldman JA, Beshansky RN, Griffin JL, Selker HP. Missed diagnose of acute cardiac ischemia in the emergency department. New Engl J Med 2000; 342: 1163-70.*
- Svensson L, Isaksson L, Axelson C, Nordlander R, Herlitz J. Predictors of myocardial damage prior to hospital admission among patients with acute chest pain or other symptoms raising a suspicion of acute coronary syndrome. Coronary artery dis 2003; 14: 225-31.*

**No series hospitalized for chest pain, n = 4**

- Lituzzo JP, Ambrose JA, Diggs P. Proton pump inhibitors for patients with coronary artery disease associated with reduced chest pain, emergency department visits, and hospitalizations. Clin Cardiol 2005; 28: 369-74.*
- Wong P, Murray S, Ramsewak A, Robinson A, van Heyningen C, Rodrigues E. Raised troponin T levels in patients without acute coronary syndromes. Postgrad Med J 2007; 83: 200-205.
- Kirk E, Prasad MK, Abdelhafiz AH. Hospital readmissions: Patient, Carer and clinician views. Acute Med 2006: 5: 104-07.
- Berkman B, Millar S, Holmes W, Bonander E. Predicting elderly cardiac patients at risk for readmission. Soc Work in Health Care 1991; 16: 21-38.
